# The fungal T-2 toxin alters the activation of primary macrophages induced by TLR-agonists resulting in a decrease of the inflammatory response in the pig

**DOI:** 10.1186/1297-9716-43-35

**Published:** 2012-04-24

**Authors:** Julie Seeboth, Romain Solinhac, Isabelle P Oswald, Laurence Guzylack-Piriou

**Affiliations:** 1 Institut National de Recherche Agronomique, Toxalim – UMR 1331, 180, chemin de Tournefeuille, Toulouse Cedex 9, 31027, France

## Abstract

T-2 toxin is known to be one of the most toxic trichothecene mycotoxins. Exposure to T-2 toxin induces many hematologic and immunotoxic disorders and is involved in immuno-modulation of the innate immune response. The objective of this work was to evaluate the effects of T-2 toxin on the activation of macrophages by different agonists of Toll-like receptors (TLR) using an in vitro model of primary porcine alveolar macrophages (PAM). Cytotoxic effects of T-2 toxin on PAM were first evaluated. An IC_50_ of 19.47 ± 0.9753 nM was determined for the cytotoxicity of T-2 toxin. A working concentration of 3 nM of T-2 toxin was chosen to test the effect of T-2 toxin on TLR activation; this dose was not cytotoxic and did not induce apoptosis as demonstrated by Annexin/PI staining. A pre-exposure of macrophages to 3 nM of T-2 toxin decreased the production of inflammatory mediators (IL-1 beta, TNF-alpha, nitric oxide) in response to LPS and FSL1, TLR4 and TLR2/6 agonists respectively. The decrease of the pro-inflammatory response is associated with a decrease of TLR mRNA expression. By contrast, the activation of TLR7 by ssRNA was not modulated by T-2 toxin pre-treatment. In conclusion, our results suggest that ingestion of low concentrations of T-2 toxin affects the TLR activation by decreasing pattern recognition of pathogens and thus interferes with initiation of inflammatory immune response against bacteria and viruses. Consequently, mycotoxins could increase the susceptibility of humans and animals to infectious diseases.

## Introduction

The Food and Agricultural Organization (FAO) estimates that mycotoxins, secondary metabolites produced by fungi, contaminate 25% of the world’s agricultural commodities. The presence of mycotoxins alters the quality of agricultural products resulting in economical losses estimated in billions dollars annually worldwide [1,2]. The consumption of food and feed contaminated by mycotoxins is a potential health hazard for both humans and animals [3,4]. Among mycotoxins, T-2 toxin is the most common trichothecene mycotoxin belonging to type A and is produced predominantly by *Fusarium sporotrichioïdes* and *F. langsethiae*[4,5]. This toxin is known to be the most cytotoxic of the trichothecene family [6]. An exposure of T-2 toxin is associated with leucopenia and cell depletion in lymphoid organs and inhibition of erythropoiesis in bone marrow and the spleen [7]. Furthermore, T-2 toxin reduces lymphocyte proliferative response [8,9] and disturbs the maturation process of dendritic cells [10] suggesting its immunosuppressant potency [7,11]. Indeed, it was previously shown that exposure to T-2 suppresses immune response to systemic infections by bacterial infection such as *Salmonella typhimurium*[12], *Listeria monocytogenes*[13], *Mycobacterium bovis*[13], *Babesia microti*[14]. Respiratory immune defences are also compromised by T-2 exposure. Indeed Li et al. [15] showed that systemic T-2 exposure increases the severity of respiratory reovirus infection with marked exacerbation of bronchopneumonia and modulation of cytokine responses in mice alveolar macrophages.

Among other species, swine react most sensitively to the exposure to trichothecenes [16]. Pigs are particularly interesting as a target species for mycotoxin due to their diet rich in cereals and because the pig shares strong similarities with the human immune system [17]. Moreover, swine are the target of several pathogenic agents responsible for many respiratory diseases such as Influenza virus, porcine reproductive and respiratory syndromes (SPRD), or bacteria as *Haemophilus parasuis*. Then, mycotoxins, especially T-2 toxin, could play a determining role in lowering the immune response of pigs to these bacterial and viral infections.

Macrophages play a key role in the immune response being the first cells to be exposed to microorganisms. These cells are considered as sentinel cells against infectious pathogens and involved in phagocytosis, antigen presentation, production of antimicrobial effector molecules and release of cytokines and chemokines that in turn contribute to immune cell recruitment and activation [18-20]. The recognition of microorganisms by macrophages occurs through a major receptor family expressed in distinct cell subsets and tissues called the Toll-Like Receptors (TLR) [21-23]. They are able to bind to a wide range of motifs derived from bacteria (lipopolysaccharide (LPS), peptidoglycan), parasites, fungi and viruses (ssRNA, dsRNA, CpG DNA) [21,24]. Interaction of TLR with its specific ligands leads to the induction of various inflammatory cytokines such as IL-1β and TNF-α [25] and reactive nitrogen species [26]. This pro-inflammatory response is the first line of defence against infections.

Since macrophages are privileged targets of T-2 toxin [10,27,28], it is important to better understand how this toxin can impair their function. In the current study, we investigated the effect of T-2 toxin on macrophage activation. We especially measured the ability of T-2 toxin to inhibit the production of inflammation mediators upon TLR activation as well as the effect of this toxin on TLR gene expression.

## Materials and methods

### Isolation, phenotype and culture of macrophages

Porcine alveolar macrophages (PAM) from 6- to 8- week-old pigs were isolated by three time bronchoalveolar lavages using cold PBS (Invitrogen, Auckland, NZ). Bronchoalveolar fluids were centrifuged at 400 *g* for 15 min at 4°C and cell viability was assessed using Trypan blue exclusion. Next, the cells were frozen in liquid nitrogen in 90% FBS and 10% DMSO. After thawing, the cells were adjusted to a cell concentration of 2 × 10^6^ cells/mL in RPMI (Invitrogen) supplemented with 10% FBS, 1% penicillin-streptomycin (Invitrogen), 1% non essential amino acid (Sigma Aldrich, Ayshire, UK) and 1% L-glutamin (Eurobio, Courtaboeuf, France).

The phenotypic identification of PAM was realized by analyzing the expression of differentiation or maturation markers: SWC1, SWC3, CD14, CD16, CD163, MHCII and DC-sign. Anti-SWC1 IgM 11-305-44 and rabbit-DC-sign antibodies were a kind gift from Dr A. Saalmüller (Veterinary University Vienna, Austria) and Dr M. Meng (College of veterinary medicine, Virginia, USA). Respectively, anti-rabbit-FITC antibody was obtained by Invitrogen. Anti-SWC3 antibodies (clones 74-22-15A and 74-22-15), CD14 (CAM36A), CD16 (G7) and MHCII (MSA3) were commercialised by VMRD (Pullman, USA). CD163 antibody (clone EDHu-1) was purchased from AbD Serotec (Oxford, UK). CD206 (clone 122D2.08) was commercialized by dendritics (Lyon, France). Reaction of all mAbs was revealed using isotype specific FITC, phycoerythrin, or biotinylated IgG F(ab)’2 fragments. Biotinylated conjugates were detected with streptavidin-Cy5-phycoerythrin. All conjugates were purchased from Southern Biotechnology Associates (Birmingham, USA). A negative control containing only conjugated-antibodies was used to visualise a non specific labelling.

PAM were incubated with different doses of T-2 toxin (Sigma Aldrich) for 1 h at 39°C in a humidified atmosphere with 5% CO_2_ and were activated by different specific TLR-agonists during 16 h at 39°C. Indeed, 39°C is the physiological temperature for pigs. Preliminary experiments indicate an optimal production of cytokines by PAM after 16 h of culture in contact with TLR-agonists. TLR-agonists were used with the following concentrations: TLR2-L (HKLM) [10 μg/mL], TLR4-L (LPS) [10 μg/mL], TLR2/6-L (FSL1) [1 μg/mL] and TLR7-L (Imiquimod) [10 μg/mL] (Invivogen, Toulouse, France). The negative control was a dilution of DMSO solution (vehicle of T-2 toxin) used at the higher concentration of T-2 toxin tested in the experiments. As a positive control of PAM activation, cells were stimulated with a combination of LPS [5 μg/mL] (Sigma Aldrich) and IFN-γ [1 ng/mL] (Biosource International, Camarillo, USA). After 4 h of activation, cells were isolated to investigate mRNA expression by quantitative real-time PCR analysis. After 16 h of activation, cells were harvested to evaluate the nitric oxide production and culture supernatant were also collected for pro-inflammatory cytokine analysis.

### Cytotoxicity, mitochondrial transmembrane potential and apoptosis assays

Cytotoxicity of T-2 toxin on PAM was measured by CellTiter-Glo luminescent cell viability assay (Promega, Madison, USA). This test is based on the quantification of the ATP assumed to be produced by metabolically active viable cells. It was used according to the manufacturer’s protocol. PAM were treated during 16 h at 39°C with different concentrations of T-2 toxin: 0.3; 1; 3; 10; 30 and 100 nM. A negative control using cells without T-2 toxin treatment was in correspondence to 100% of cell viability. All experiments were performed in triplicate and repeats on cells came from three different piglets. IC_50_ was determined with SigmaPlot software for windows version 11.0 (Systat software Inc., Chicago, USA).

For mitochondrial transmembrane potential (ΔΨ_m_) measurement, cells were treated for 16 h with the same range of T-2 toxin concentrations as previously described. DIOC_6_ probe obtained by Invitrogen was added to 100 μL of cell suspension and was incubated at 39°C for 20 min. ΔΨ_m_ was analyzed by flow cytometry with excitation at 488 nm. A negative control without T-2 toxin and a positive control, FCCP (Carbonyl cyanid 4-trifluoromethoxy-phenylhydrasone, Enzo Life Sciences,Villeurbanne, France) at 40 mM were used to determine fluctuation of ΔΨ_m_.

Apoptosis was determined by AlexaFluor488-AnnexinV/Death cell apoptosis kit (Invitrogen). The cells were collected, and 2 × 10^6^ cells per mL were resuspended in PBS-1X (pH 7.4) and incubated with AlexaFluor488annexin-V and propidium iodide (PI). The staining was realized according to the manufacturer’s recommendation.

Flow cytometric acquisition was performed on a flow cytometer (FACS Calibur, Becton Dickinson, Franklin Lakes, NJ, USA) using CellQuest software (Becton Dickinson) and FlowJo software for analysis (Ashland, USA).

### IL-1β and TNF-α cytokine assays

Concentrations of IL-1β and TNF-α were measured by enzyme linked immuno-absorbent assays (ELISA) using kits specific for porcine IL-1β and TNF-α (R&D Systems, Minneapolis, MN, USA). The plates were washed with PBS/Tw20 and then blocked with PBS containing 1% BSA (w/v) for 1 h at RT. The test supernatants were added to the ELISA plate in duplicate and incubated for 2 h at RT. After washing, the wells were incubated with the biotinylated detection anti-porcine TNF-α/TNFSF1A and anti-porcine IL-1β/IL-1 F2 (R&D Systems) antibodies for 2 h at RT. Then, streptavidin-HRP-conjugated antibody (Thermo Fisher Scientific, Courtaboeuf, France) was added for 30 min at RT. Positive reactions were revealed by TMB, a colour substrate (Thermo Fisher Scientific) and reactions were stopped with H_2_SO_4_ 2 N. The OD was read at 450 nm. A negative control was in correspondence to non-activated TLR-agonist cells and as a positive control, cells were stimulated with a combination of LPS [5 μg/mL] and IFN-γ [1 ng/mL]. Concentrations obtained in negative control wells were subtracted from the values in treated samples.

### Nitric oxide assay

Nitric oxide measurement was performed by DAF-FM diacetate (4-amino-5-methylamino-2′,7′-difluorofluorescein diacetate) probe. The probe used a non-fluorescent reactive base able to react to low doses of nitric oxide forming a fluorescent compound which is detectable by flow cytometry. DAF-FM was used at 10 μM according to the manufacturer’s protocol (Invitrogen). After DAF-FM addition, PAM were incubated at 39°C for 30 min then washed twice with PBS. Cells were incubated another time during 15 min at 39°C to allow a complete esterification of intracellular diacetate and the cell fluorescence emission producing nitric oxide were analyzed by flow cytometry. A negative control was in correspondence to non-activated TLR-agonist cells and as a positive control cells were stimulated with a combination of LPS [5 μg/mL] and IFN-γ [1 ng/mL]. Concentrations obtained in the negative control, including no T-2 toxin and no stimulation with any TLR agonist treatments were subtracted from the values obtained with treated cells.

### RNA extraction and quantitative real-time polymerase chain reaction (PCR) analysis

Quantitative real-time PCR was performed to determine the relative TLR −2, -4, -2/6, -7 and iNOS (inducible Nitric Oxide Synthase) mRNA expression levels. Total RNA from 2 × 10^6^ cells/mL unstimulated cells and 2 × 10^6^ cells/mL ligand-stimulated cells in the presence or absence of T-2 toxin was extracted with TRIzol Reagent (Extract all, Eurobio). Concentrations, integrity and quality of RNA were determined spectrophotometrically (O.D._260_) using Nanodrop ND1000 (Labtech International, Paris, France). Then, 300 ng of RNA was reverse transcribed and the reverse transcription and real-time PCR steps were performed as previously described [29]. Non-reverse transcribed RNA was used as the non-template control for verification of a no genomic DNA amplification signal. Specificity of PCR products was assessed at the end of the reaction by analyzing the curve of dissociation. Primers were purchased from Invitrogen. Primers used for real time quantitative polymerase chain reaction are described in Table 1[30-34]. Amplification efficiency and initial fluorescence were determined by the DART-PCR method, then the values obtained were normalized with housekeeping genes, ribosomal protein L32 (RPL32) and beta2-microglobulin. Finally, relative gene expression was expressed in comparison to the control culture corresponding to unstimulated cells.

**Table 1:** **Primers used for real time quantitative polymerase chain reaction.** List of gene sequences and primers (For: Forward; Rev: Reverse) used for real-time qPCR of immune-related genes

Oligo Names	Medline mRNA Sequence	Sequence 5′ → 3′	Reference
β2-microglobuline For β2-microglobuline Rev	MN_213978	TTCTACCTTCTGGTCCACACTGA TCATCCAACCCAGATGCA	[34]
RPL32 For RPL32 Rev	MN_001001636	TGCTCTCAGACCCCTTGTGAAG TTTCCGCCAGTTCCGCTTA	[32]
iNOS For iNOS Rev	EST BI344008	GAGAGGCAGAGGCTTGAGAC TGGAGGAGCTGATGGAGTAG	[30]
TLR2 For TLR2 Rev	AB085935	TCACTTGTCTAACTTATCATCCTCTTG TCAGCGAAGGTGTCATTATTGC	[33]
TLR4 For TLR4 Rev	AB188301	GCCATCGCTGCTAACATCATC TCAGCGAAGGTGTCATTATTGC	[33]
TLR6 For TLR6 Rev	AB085936	AACCTACTGTCATAAGCCTTCATTC GTCTACCACAAATTCACTTTCTTCAG	[33]
TLR7 For TLR7 Rev	AB258453	CAGAAGTCCAAGTTTTTCCAGCTT GGTGAGCCTGTGGATTTGTTG	[31]

### Statistics

A Fisher’s test was realized followed by the Student’s *t-*test to compare values between multiple groups. The statistical analysis of the data was carried out with Statview software (Statistical Analysis System; SAS for Windows 98; SAS Institute Inc., Cary, NC, USA). A *P* value of less than 0.05 was considered to be statistically significant.

## Results

### Phenotypic characterization of primary porcine alveolar macrophage cells (PAM)

The specific staining with different markers confirmed that more than 95% of the cultured cells were PAM displaying the following phenotypes: SWC3^high^, SWC1^+^, CD163^high^, CD14^-^, CD16^+^, MHCII^+^ and DC-sign^+^ ( Additional file 1: Figure S1).

### Cytotoxicity effects of T-2 treatment on PAM

**Figure 1:**
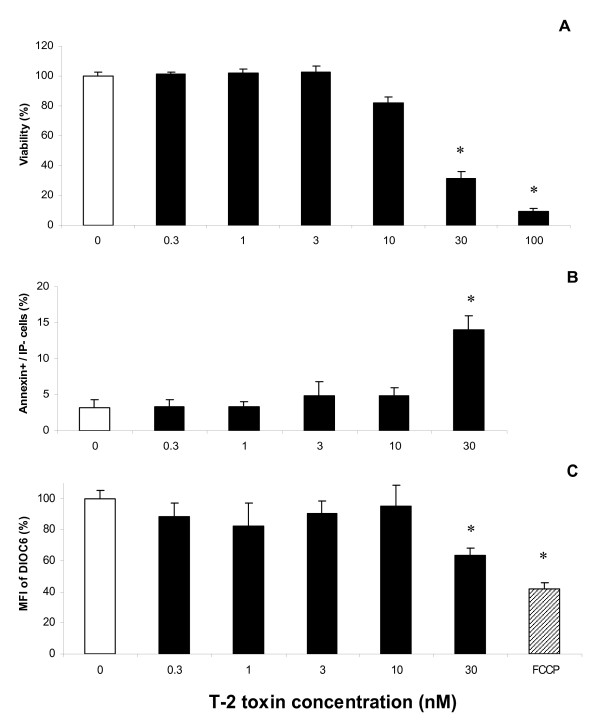
**Cell viability and apoptosis induced by T-2 toxin in porcine alveolar macrophages (PAM).** A-Decrease of cell viability in PAM treated with different T-2 toxin concentrations. PAM cultures were exposed to 0.3–100 nM of T-2 toxin for 16 h. Cell viability was assessed by measurement of ATP release. B- Increase of apoptosis in PAM as a function of different T-2 toxin concentrations. PAM cultures were exposed to 0.3 to 30 nM of T-2 toxin. Apoptotic cells were analyzed by flow cytometry using a double staining AnnexinV/IP. C- Decrease of mitochondrial membrane potential (Δψm) in PAM treated with T-2 increasing toxin concentrations. PAM cultures were exposed to 0.3 to 30 nM of T-2 toxin, followed by flow cytometry analysis of DIOC6 probe. A positive control, FCCP (Carbonyl cyanide 4-(trifluoromethoxylphenylhydrazone)) was used to establish a loss of mitochondrial membrane potential (Δψm). All data were reported to the negative control including only the vehicle (0-DMSO), used as 100% of cell viability. Data are shown as mean +/− SEM with PAM from three different piglets are shown and were performed in triplicate. * indicates a *P* value < 0.05.

A dose-dependent effect of T-2 toxin on cell viability was demonstrated by measuring the cellular ATP level after exposing PAM to increasing concentrations of the toxin (Figure 1a). T-2 toxin did not induce a cytotoxic effect on PAM from 0.3 to 3 nM while exposure to 10 nM of T-2 toxin reduced the ATP level to 82% compared to the untreated cells. At 30 nM, T-2 toxin induced a dramatic drop of cell viability leading to only 31.4% of ATP level compared to the control. IC_50_ value deducted from the dose–response curve was estimated at 19.47 nM +/− 0.9753 nM.

To determine if the loss of cell viability is a consequence of an apoptotic process, the identification of early apoptotic cells was measured using FITC-annexinV reagent in conjunction with a vital dye, propidium iodide (PI). T-2 toxin (<10 nM) did not induce apoptosis (Figure 1b). Only concentrations over 30 nM produced a significant increase of Annexin^+^/PI^-^ cells (14%), revealing apoptosis of macrophages.

Apoptosis is associated with a loss of mitochondrial membrane potential (Δψm). To confirm the loss of viability induced by T-2 toxin (Figure 1a and b), a DIOC6 staining which is a mitochondrial-specific and voltage-dependent dye was carried out (Figure 1c). As expected, treatment by FCCP, a potent reversible inhibitor of mitochondrial oxidative phosphorylation, used as a positive control, induced a significant decrease of 41.6% of the DIOC6 signal. From 0.3 to 10 nM, T-2 toxin did not affect Δψm, while 30 nM of T-2 toxin significantly reduced Δψm of PAM (63.6% of MFI compared to the control). Thus, DIOC6 staining results confirmed the induction of the apoptosis by high dose of T-2 toxin and demonstrated the similar dose–response regarding ATP level and mitochondrial membrane potential. Based on these results, a concentration of 3 nM was chosen for the following studies.

### Alteration of macrophage activation by T-2 toxin

**Figure 2:**
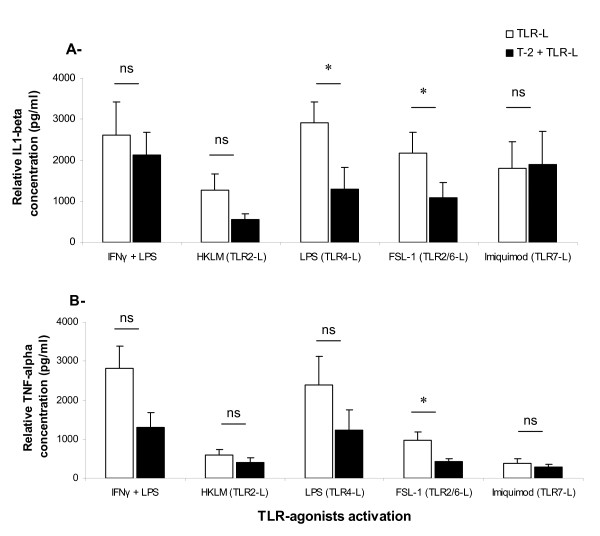
**Concentration of IL-1β and TNF-α in supernatants of PAM pre-incubated with T-2 toxin.** Cells were cultured for 1 h in the presence (black bars) or absence (white bars) of 3 nM of T-2 toxin before their activation with different TLR-agonists: TLR2-agonist (HKLM = 10 μg/mL); TLR4-agonist (LPS = 10 μg/mL); TLR2/6-agonist (FSL1 = 1 μg/mL) and TLR7-agonist (Imiquimod = 10 μg/mL) for 16 h. IL-1β (A) and TNF-α (B) concentrations were determined by ELISA in PAM culture supernatants. Concentrations obtained in negative controls (vehicle) were subtracted from concentration values in treated samples. The mean (+/− SEM) of the values obtained with PAM from five different piglets are shown. * indicates a *P* value < 0.05.

To investigate the effects of T-2 toxin on macrophage activation, the cells were exposed for 1 h to 3 nM of T-2 toxin before being treated with different TLR-agonists during 16 h (TLR2, TLR4, TLR2/6 and TLR7 agonists). Two cytokines involved in the inflammation process were measured to determine the macrophage activation status: IL-1β and TNF-α. The positive control (combination of LPS and IFN-γ) led to high production of both IL-1β and TNF-α, at levels similar to those found with TLR4 agonist (Figure 2a and b). Basal levels of both IL-1β and TNF-α concentrations obtained in the negative control including neither T-2 toxin nor stimulation with TLR-agonists were set off. However, cytokine levels were different depending on the nature of TLR-agonists, demonstrating a specific ability of each TLR-agonist to induce a particular level of cytokine responses in PAM. In particular, TLR2, TLR2/6 and TLR7 agonists seem to induce lower production of TNF-α compared to TLR4 and IFN-γ/LPS (Figure 2b).

Pre-exposure to 3 nM of T-2 toxin significantly decreased both IL-1β and TNF-α induced by TLR2/6 agonist (50.4% and 55.9% reduction of production respectively) and only a significant decrease of IL-1β induced by TLR4 agonist (55.6%). Interestingly, pre-treatment with T-2 toxin did not affect the production of IL-1β and TNF-α cytokines in response to TLR7-agonist activation (Figure 2a and b).

### Alteration of iNOS gene expression and nitric oxide production induced by macrophage activation after T-2 toxin treatment

**Figure 3:**
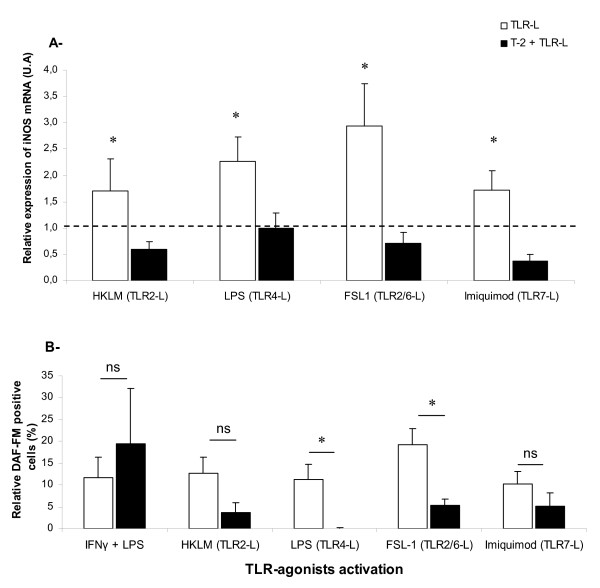
**Analysis of relative expression of iNOS mRNA (A) and nitric oxide (NO) production (B) in PAM pre-treated with T-2 toxin.** A- T-2 toxin affects iNOS mRNA expression: Relative mRNA expression of iNOS in PAM treated or not by 3 nM of T-2 toxin for 1 h before their activation with each specific TLR-agonist during 4 h. Data were analyzed with DART-PCR software and iNOS expression was normalized by the mean of two housekeeping genes (β2-microglobuline and RPL32). Gene expression of iNOS was reported to unstimulated PAM whose expression is visualized by the line level at 1. Significant differences (*P* < 0.05) between unstimulated PAM (negative control) and cells treated (with TLR-agonists in the absence or presence of T-2) are marked with an asterisk. B- T-2 toxin affects NO production: the cells were cultured for 1 h in the presence (black bars) or absence (white bars) of 3 nM of T-2 toxin before their activation with different TLR-agonists: TLR2-agonist (HKLM); TLR4-agonist (LPS); TLR2/6-agonist (FSL1) and TLR7-agonist (Imiquimod) for 16 h. An incubation of both cells and DAF-FM probe was used to determine the production of NO. Mean Fluorescence Intensity (MFI) of DAF-FM +/− SEM was determined by flow cytometry analysis and then normalized with the negative control. Mean (+/− SEM) of values obtained with PAM from five different piglets are shown. * indicates a *P* value < 0.05.

Nitric oxide (NO) production plays an important role in the protection against intracellular microbiostasis. NO, produced by macrophages in response to specific stimuli is catalyzed by inducible Nitric Oxide Synthase (iNOS). Relative gene expression of iNOS was assessed by quantitative real time PCR (Figure 3a). As expected, TLR-agonists induced a significant increase of iNOS expression. By contrast, pre-treatment with T-2 toxin abrogated expression of iNOS induced by all TLR-agonists used. Relative gene expression of iNOS was closer than the expression of negative controls. To confirm these results,the inhibition of NO production in activated PAM by a T-2 toxin pre-treatment was investigated. The production of NO was measured by flow cytometry, using the DAF-FM probe (Figure 3b). As observed for pro-inflammatory cytokines, IFN-γ/LPS combination as well as TLR-agonist stimulation led to NO production by macrophages, thereby confirming the activation process. However, T-2 pre-treatment did not seem to affect production of NO by IFN-γ/LPS co-stimulation. In addition, T-2 toxin pre-treatment significantly decreased to 100 and 72.5% the NO production after activation by TLR4 and TLR2/6 agonists respectively. T-2 toxin pre-exposure has also non-significantly decreased (*p* = 0.06 and *p* = 0.32) the production of NO initiated by the ligands of both TLR2 and −7 (69.8 and 49.0% reduction of production). These data suggest that T-2 treatment alters the implementation of pro-inflammatory response which is initiated by TLR-agonists. Transcriptomic data of iNOS gene expression could partially explain the decrease of NO production by porcine alveolar macrophages observed with DAF-FM staining.

### Effects of T-2 toxin on mRNA expression of TLR

To understand the decrease of the pro-inflammatory response, the effect of T-2 toxin on the mRNA expression of specific TLR was evaluated. As expected, treatment of PAM by specific TLR-agonists, led to significant increase in relative mRNA expression of specific TLR (Table 2). It is worth mentioning that T-2 pre-exposure did not lead to a complete inhibition of the mRNA expression. However, a pre-incubation with 3 nM of T-2 toxin before activation induced a significant decrease of 60.1%, 64.3% and 68.8% of relative expression of TLR2, TLR4 and TLR2/6 mRNA expression respectively (Table 2). Remarkably, T-2 toxin did not significantly reduce mRNA expression of TLR7.

**Table 2:** Relative mRNA expression of TLR (−2, -4, -2/6 and −7) after T-2 toxin treatment in PAM

	negative control	TLR-L	T-2 + TLR-L
HKLM (TLR2-L)	1 +/− 0.14	76.92 +/− 16.67*	29.08 +/− 8.16 (*), (t)
LPS (TLR4-L)	1 +/− 0.11	17.69 +/− 4.80*	6.32 +/− 1.46 (*), (t)
FSL1 (TLR2/6-L)	1 +/− 0.22	259.90 +/− 75.91*	81.07 +/− 15.62 (*), (t)
Imiquimod (TLR7-L)	1 +/− 0.20	13.66 +/− 3.92*	7.03 +/− 1.66 (*)

Primary porcine cells (2 × 10^6^ cells per mL) were exposed or not with 3 nM of T-2 toxin for 1 h before activation during 4 h at 39°C by different TLR-agonists: TLR2-agonist (HKLM); TLR4-agonist (LPS); TLR2/6-agonist (FSL1) and TLR7-agonist (Imiquimod). Relative expression of TLR genes was evaluated by quantitative real time PCR. Data were analyzed by DART-PCR software and gene expression was normalized by the mean of two housekeeping genes (RPL32 and β2-microglobuline). TLR gene expression was reported to unstimulated PAM. After checking for the normal distribution of data carried out with one-way ANOVA test, Student test was conducted to determine the differences among groups. The mean (+/− SEM) of the values obtained with PAM from five different piglets are shown. Significant differences (*P* < 0.05) between unstimulated cells (negative control) and cells treated by each TLR-L are marked with an asterisk (*). Significant differences (*P* < 0.05) between TLR-L-activated cells in the presence or not of T-2 toxin treatment are marked with (t).

### Effects of T-2 toxin on the expression of cell surface receptors of macrophages

**Figure 4:**
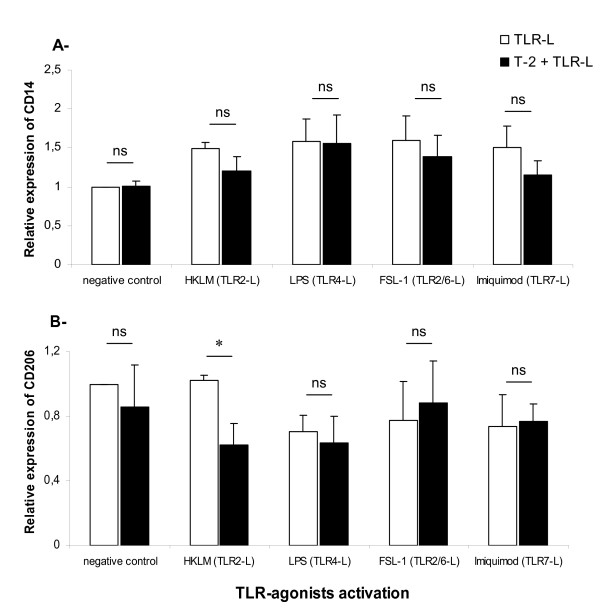
**Effects of T-2 toxin on the expression of both cell surface receptor on macrophage: TLR4 co-receptor (CD14) and mannose receptor (CD206).** Cells were cultured or not for 1 h in the presence (black bars) or absence (white bars) of 3 nM of T-2 toxin before their activation with different TLR-agonists: TLR2-agonist (HKLM); TLR4-agonist (LPS); TLR2/6-agonist (FSL1) and TLR7-agonist (Imiquimod) for 16 h at 39°C, 5% CO_2_. Cells were collected and CD14 (A) and CD206 (B) staining were analyzed by flow cytometry. The mean of fluorescence intensity of both of these two markers was normalized to the negative control. Mean (+/− SEM) of values obtained with PAM from five different piglets are shown. * indicates a *P* value < 0.05

In order to examine the specificity of action of T-2 toxin on cell surface receptors, we analyzed the expression by flow cytometry of two receptors, namely CD14 (co-receptor with the TLR4 for the detection of bacterial LPS) and CD206 (macrophage mannose receptor) present on the cell surface, after treatment with T-2 toxin (Figure 4). Surprisingly and in contrast to the T-2 toxin action observed on TLR expression, the expression level of both receptors was not affected by the presence of T-2 toxin (Figure 4). Moreover, TLR-agonist stimulation did not induce an increase of expression of these two cell surface markers.

## Discussion

Pigs are preferential targets of many respiratory diseases caused by bacteria such as *Pasteurella multocida*[35]. Different swine respiratory viruses such as African swine fever virus (ASFV) [36] or porcine reproductive and respiratory syndrome virus (PRRSV) [37] also target alveolar macrophages for their replication. It is well established that when incorporated in feed or given in daily doses, T-2 toxin decreases resistance against viral or bacterial infections [12,15]. Moreover, Ziprin et al. [13] showed that the T-2 toxin effect on the course of in vivo bacterial infection depends on the nature of the infectious agent. Based on these different data, the present study is of further interest to address whether T-2 toxin can similarly reduce immune resistance depending on the nature and composition of microbial pathogens, using TLR4 and TLR6-agonists, structurally conserved molecules derived from these pathogens.

The current study indicates that the immune response implemented by alveolar macrophages is impaired by T-2 toxin. T-2 toxin is known to be cytotoxic and to induce apoptosis on immune cells like human monocytes, human dendritic cells and human and rat macrophages [10,28,38,39]. We observed that after 16 h exposure to T-2 toxin, the cell viability of PAM is affected by 10 nM of T-2 toxin and the IC_50_ value was evaluated near 20 nM. This concentration was in accordance with other studies on immune cells [10,40]. Cell death may be due to necrosis or apoptosis. The T-2 toxin is known to induce the apoptosis pathway at varying concentrations depending on the targeted cells [38,39,41,42]. For example, in primary culture cells of hematopoietic progenitors, T-2 toxin induces apoptosis after 3 h of incubation with 10 nM of the toxin and a maximum reached at 12 h [43] while in macrophage cell lines (RAW 267.7), T-2 toxin induces less than 5% of apoptotic cells after 6 h of incubation with 5 nM of T-2 toxin [44]. The latter results are comparable to our data. A significant induction of apoptosis was shown with the exposure of 30 nM of T-2 toxin on PAM after 16 h of treatment. In order to avoid possible adverse effects due to cytotoxic and apoptotic process, a non cytotoxic and non apoptotic concentration of 3 nM of T-2 toxin was used for PAM activation studies. Moreover, the dose of 3 nM of T-2 toxin was relevant as a low dose and close to the provisional tolerable daily intake (TDI) of 100 ng / Kg bw / d [4].

Activated macrophages are the primary source of pro-inflammatory cytokines, including IL-1β and TNF-α [27,45]. Production of these two cytokines, in response to TLR-agonists and notably LPS, could be observed in the present study. Interestingly, after pre-treatment with low doses of T-2 toxin, a reduction of pro-inflammatory cytokine concentrations appeared dependent on the nature of the TLR-agonists. The IL-1β production was reduced after activation by the TLR2-agonist and significantly decreased after TLR4- and TLR6-agonist activation. By contrast, we did not observe a reduction of IL-1β production after activation of Pathogen Associated Molecular Patterns (PAM) by the TLR7-agonist. Similar results were observed regarding TNF-α response.

TLR expressed on antigen presenting cells (APC) such as macrophages, are critical for recognition of the PAMP on microorganisms and their abilities to induce appropriate immune responses. Extensive research on TLR has been performed in humans and rodents while only a few studies on porcine TLR have been completed. However, most of the studies on swine TLR have clearly demonstrated a correlation between the expression levels of TLR2 and TLR9 expressed in several tissues (lung, heart, spleen, small intestine, mesenteric lymph nodes…) at the first stages of life and the capacity for newborn piglets to have an early efficient immune function [46,47]. Moreover, similar distribution of TLR on human and swine cells, unlike rodents, has been previously described [48]. Human alveolar macrophages have low TLR2 expression leading to a low production of TNF-α and IL-6 cytokines [49]. Similarly, the TLR2 expression level measured in human bronchoalveolar cells by Juarez et al. [49] could explain the low TNF-α protein concentration obtained in response to the TLR2-agonist in this study.

It is known that activation of macrophages by agonists, such as LPS, triggers the enhancement of expression of iNOS leading to NO production [26]. In the present investigation, a T-2 toxin pre-treatment induced a significant decrease of NO production in PAM activated by TLR4 and TLR2/6 agonists. However, this toxin did not affect NO production after activation of macrophages by either IFN-γ/LPS or TLR2 and −7 agonists. DON and nivalenol also affect the immune response by inhibiting NO production in mice macrophage cell lines in vitro (RAW264) [50]. In addition, this study demonstrated that there is a parallel between the decreasing of NO production and the decreasing of iNOS mRNA gene expression which could partially explain the effect of T-2 toxin on NO production.

Taken together, these results demonstrate the complexity of mycotoxin action depending on the cell activation status and mycotoxin doses. The TLR agonist-sensitized macrophage responds to subsequent toxin challenge with a robust and potentiated cytokine response. By contrast, our study showed that T-2 toxin pre-exposure inhibited naive macrophage to initiate an inflammatory response to TLR-ligands. This inhibition could be attributed to a defect of pathogen pattern recognition due to a T-2 toxin effect on TLR2, -4 and −2/6 expression. Due to a lack of commercially available antibodies for pigs, we could not verify the cell surface expression of these TLR. However, the present study showed that the importance of this inhibition seems to depend on the nature of the TLR. Indeed, the expression of TLR7 mRNA seemed less affected by the toxin. These results were consistent with the less inhibitory effect of T-2 toxin on the inflammatory response when PAM were activated by TLR7-agonist. This one is specifically located on the endosomal membrane and could explain that a T-2 toxin pre-exposure did not appear to disrupt the pro-inflammatory response.

The alteration of TLR expression could possibly influence the functions and efficacy of alveolar macrophages in terms of antigen processing and release of cytokines. It is tempting to speculate that the sensitized macrophage phenotype depends on both appropriate surface TLR expressions enabling the recognition of pathogen motifs and the appropriate induction of the cytokine signaling pathway. However, the effects of T-2 toxin seem to be TLR-receptor specific since neither co-receptor CD14 nor mannose receptor (CD206) were affected by the toxin. Our group has already shown in a previous study that DON can also inhibit cell surface expression of activation markers of human macrophages such as CD14, CD54, CD119 and HLA-DR [51].

Trichothecenes have the ability to modulate immune function by disrupting intracellular signaling pathways within leukocytes responsible for driving both altered immune-regulatory gene expression as well as apoptosis [52]. A review by Pestka et al. [53] demonstrated the preferential targeting of DON on the activation of mitogen-activated protein kinases (MAPK), thereby leading to the modulation of immune responses and apoptosis [53]. In addition to specific intracellular localization of TLR7, the absence of T-2 toxin action on immune responses after TLR7 agonists activation can be explained by focusing on the specificity of TLR7-signaling pathways. In contrast to other TLR which involved both MAPK and canonical IKK complex for the transcription of inflammatory cytokine genes, TLR7 agonist activates only the IKKα signaling pathway to induce inflammatory cytokines [21].

Based on our finding that the T-2 toxin down-regulated the activation of TLR, it could be hypothesized that exposure to low concentrations of T-2 toxin may increase the susceptibility of humans and animals to opportunistic infections. The present study sheds new light on the potential of mycotoxins to interfere with the immune system by decreasing the expression pattern recognition of pathogens, and thus the initiation of immune responses against bacterial and viral infections. These results may help to explain the immunosuppressive effect of T-2 toxin observed in vivo during viral respiratory infection such as reovirus [15].

Given that food and feed are sometimes contaminated by T-2 and other trichothecenes, serious questions remain regarding risks from chronic ingestion of these toxins. Understanding the molecular mode of action of a mycotoxin can assist in predicting potential adverse human and animal health effects.

## Abbreviations

APC = Antigen presenting cells; ATP = Adenosine-5′-triphosphate; CD = Cluster of differentiation; DAF-FM diacetate = 4-amino-5-methylamino-2′,7′-difluorofluorescein diacetate; DON = Deoxynivalenol; FCCP = Carbonyl cyanid 4-trifluoromethoxy-phenylhydrasone; IFN-γ = Interferon gamma; IL-1β = Interleukine 1 beta; iNOS = Induce nitric oxide synthase; LBP = LPS-binding protein; LPS = Lipopolysaccharide; MAPK = Mitogen-activated protein kinases; mRNA = Messenger ribonucleic acid; MyD88 = Myeloïd differentiation primary response gene (88); NIV = Nivalenol; NO = Nitric oxide; PAM = Porcine alveolar macrophages; PAMP = Pathogen-associated molecular patterns; PCR = Polymerase chain reaction; PI = Propidium iodide; PRRs = Pattern recognizing receptors; RNA = Ribonucleic acid; SPRD = Porcine reproductive and respiratory syndrome; ssRNA = Single strand RNA; TNF-α = Tumor necrosis factor alpha; TLRs = Toll-like receptors; TLR-L = Toll-like receptors ligand or agonist.

## Competing interests

The authors declare that they have no competing interests.

## Authors’ contributions

JS contributed to the acquisition, the analysis, the interpretation of cytotoxicity, transcriptomic and proteomic data and prepared the manuscript draft. RS has to take part in the design of this research and he carried out the statistical analysis. IPO contributed to the design but also took part in the drafting of the article and the critical revision of the important intellectual contents. LGP contributed to the acquisition, the analysis and the interpretation of flow cytometry, transcriptomic and proteomic data as well as coordination of the study and preparation of the manuscript. All authors read and approved the final manuscript.

## Supplementary Material

Additional file 1 **Figure S1.** Morphologic and phenotypic characterization of PAM by flow cytometry using SWC3, SWC1, CD163, CD14, CD16, MHCII and DC-sign staining. This figure is a representative of three independent experiments. Click here for file
