# Supplementary figures and images for: The fungal T-2 toxin alters the activation of primary macrophages induced by TLR-agonists resulting in a decrease of the inflammatory response in the pig
Source: Vet Res. 2012 Apr 24;43(1):35. doi: 10.1186/1297-9716-43-35 (PMC3416672; doi:10.1186/1297-9716-43-35)

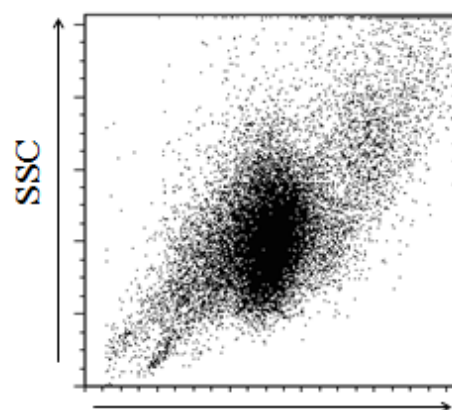

FCS

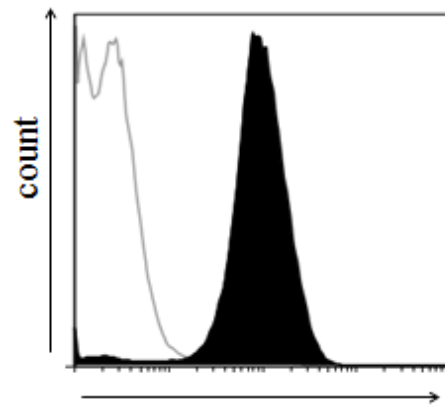

SWC3

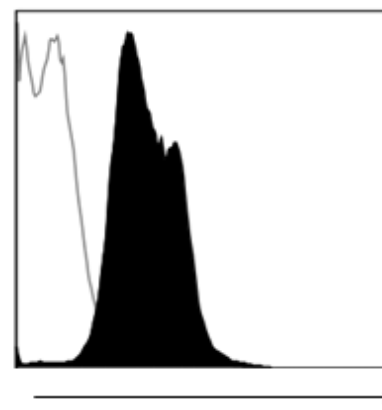

SWC1

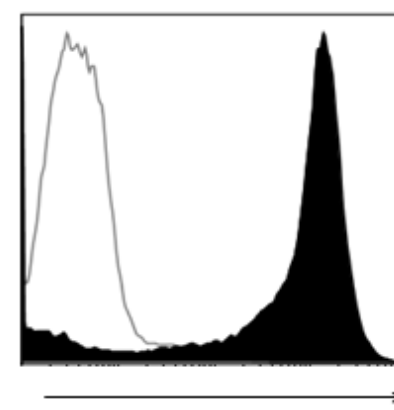

CD163

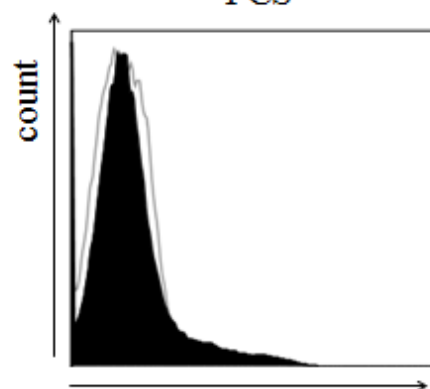

CD14

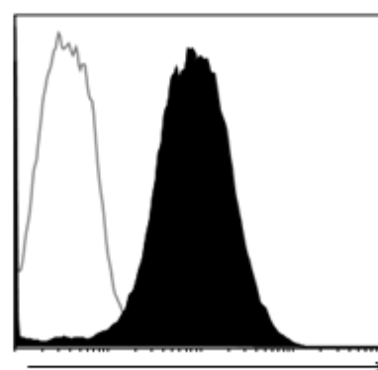

CD16

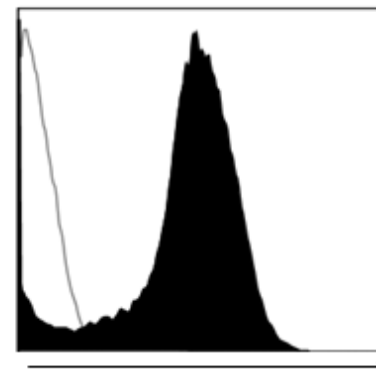

MHCII

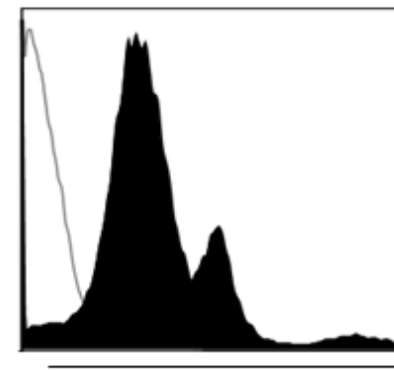

DC-sign

Supplement: Additional file 1 — Figure S1. Morphologic and phenotypic characterization of PAM by flow cytometry using SWC3, SWC1, CD163, CD14, CD16, MHCII and DC-sign staining. This figure is a representative of three independent experiments. [file 1297-9716-43-35-S1.pdf]
